# Assessment of doped graphene in the removal of atrazine from water

**DOI:** 10.1038/s41598-024-71886-2

**Published:** 2024-09-23

**Authors:** Ahmed Hellal, Hazem Abdelsalam, Walid Tawfik, Medhat A. Ibrahim

**Affiliations:** 1 Department of Laser in Meteorology, Photochemistry and Agriculture (LAMPA), National Institute of Laser Enhanced Sciences, Cairo University, Giza, 12613 Egypt; 2 School of Materials Science and Engineering, Yancheng Institute of Technology, Yancheng, 224051 People’s Republic of China; 3 Theoretical Physics Department, National Research Centre, El-Buhouth Str., Dokki, Giza, 12622 Egypt; 4 Spectroscopy Department, National Research Centre, Giza, 12622 Egypt; 5 Molecular Modeling and Spectroscopy Laboratory, Centre of Excellence for Advanced Science, National Research Centre, 33 El-Bohouth St., Dokki, Giza, 12622 Egypt

**Keywords:** Graphene quantum dots, AHEX, Atrazine, Adsorption properties, DFT, Simulation, Water contamination, Pollution remediation, Ecological modelling, Mechanical and structural properties and devices

## Abstract

Atrazine is a widely used toxic herbicide that poses a threat to both the environment and human health. This study investigates the removal of Atrazine from water through armchair-hexagonal hexagonal graphene quantum dots (AHEX) simulations. The investigations are performed using density functional theory at the exchange–correlation hybrid functional B3LYP/3-21G level of theory. The activity of pristine AHEX, with a total dipole moment of 0.0 (debye), is enhanced by doping with boron (B), nitrogen (N), and sulfur atoms (S), resulting in increased total dipole moments of 8.99, 5.29, and 4.14 Debye respectively. This enhancement occurs without any structural deformation due to the doping process. Our results show significant adsorption capacity of the doped nanographene for Atrazine, evidenced by the high adsorption energies of 0.52 eV for boron, 0.62 eV for nitrogen, and 2.97 eV for sulfur. Charge distribution on the atrazine complexes further confirms effective interaction, with values of 0.03,  − 0.018, and 0.032 (e). UV–vis spectroscopy reveals that the prominent absorption peaks of boron and nitrogen-doped samples, initially at ~ 658.8 and 431 nm, undergo a redshift to ~ 676 and 444.3 nm after adsorption, respectively. This redshift aligns with the dominant excitation moving to lower energies following adsorption. Conversely, the sulfurated nanographene shows a blue shift from 980.66 to 485.41 nm. These findings highlight the potential of doped nanographene as an effective treatment for atrazine-contaminated water.

## Introduction

Graphene doping refers to introducing foreign atoms into the graphene lattice to modify its electronic characteristics^1^. The band structure of graphene can be modulated by substitutional doping, according to many theoretical investigations^2–4^. Typically, doping graphene involves two methods: (I) the materials are adsorbed onto the surface, which may be gas^5^, metal^6^, or organic^7^, and (II) some atoms get doped into the lattice structure of graphene^8^. Graphene can be doped with a variety of elements, including P-Block in the periodic table with good electronegativity elements like nitrogen (N), sulfur (S), boron (B), phosphor (P), oxygen (O), and fluorine (F)^9–11^. Recently, the practice of doping graphene with heteroatoms such as nitrogen (N), Phosphorus (P), Boron (B), and Sulfur (S) has garnered significant interest^12^. This chemical modification of graphene alters its properties, opening up various potential applications^13^. These include uses in water purification, energy storage, biomarker detection, catalysis, and various other fields^14–16^. Fortunately, heteroatom doping of graphene can provide many active sites for the material. Furthermore, its adaptable nature can be utilized in composite materials since chemical changes make it easier to tweak its surface qualities in an advantageous way^17,18^. When compared to pristine graphene, the N-graphene exhibits distinct features. For example, the neighboring nitrogen dopants will affect carbon atoms' spin density and charge distribution^19^. Doping creates an “active region” on the surface of graphene. Additionally, following the introduction of nitrogen doping into monolayer graphene, the Fermi level is elevated above the Dirac point^20^. This means fewer energy states are available after doping (with nitrogen) for the electrons to occupy near the Fermi level. The suppression of the density of states near the Fermi level leads to the opening of the band gap^1^. Through p- or n-type doping, the Fermi level can be changed in the conduction- or valence band and the band gap can even be opened, depending on the addends^21,22^. The production pathway selection is the first step towards a successful functionalization of graphene since it establishes constraints for modifying the carbon lattice.

Density functional theory (DFT) by (Iqbal et al. 2021)^23^ states that there are four ways that sulfur atoms might appear in doped graphene: adsorption to the surface, substitution of carbon atoms at the graphene edge, production of S/S oxides, and formation of sulfur clusters to create a ring connecting two graphene layers. Sulfur is more likely to replace graphene’s carbon atoms in a serrated edge to achieve doping from an energy perspective^24,25^.

Table 1 summarizes past studies demonstrating that doped graphene materials incorporating heteroatoms such as nitrogen (N), phosphorus (P), sulfur (S), and boron (B) have shown improved adsorption efficiency compared to pristine graphene.

**Table 1:** Adsorption efficiencies of heteroatom-doped graphene materials to adsorb organic material.

No.	Doped material	Adsorbate	Adsorption capacity	References
1	N-doped graphene aerogel	Methylene Blue Dye	216.96 mg/g	Ding et al.^26^
2	N-S Co-doped graphene (with Dithiobisurea)	Cadmium	1.755 mmol/g	Kong et al.^27^
3	Graphene-boron nitride composite	Ciprofloxacin	185 mg/g	Han et al.^28^
4	B-doped graphene	Hydrocarbon oil	72 g/g	Gupta^29^

The extensive use of herbicides endangers human and other species' health by contaminating the world's water supplies. The EU's drinking water directive permits herbicide concentrations between 0.1 and 0.5 μg L^−1^^30,31^. High atrazine levels in water bodies result from volatilization during spraying, surface runoff, soil leaching, accidental releases, manufacturing emissions, and improper disposal. atrazine's long-term persistence was evidenced by detection levels of 0.1–3.4 μg kg^−1^ in soils 22 years post-application and 19.5 μg kg^−1^ in agricultural soil after 20 years^32,33^. Between 1 and 6% of herbicides reach aquatic environments through leaching and runoff from forests and agricultural lands, with groundwater contamination exacerbated by insufficient organic carbon and microorganisms for breakdown^34^. Water treatment is essential to ensuring that water resources are clean and safe for consumption^35^ Adsorption, photocatalysis, and electrochemical reactions are the main techniques of Gr-based materials used to remove various toxins from wastewater^36^. Water treatment was also tried via other common techniques. In this session, the effects of Na^+^/H_2_O_2_ coexistence on sludge denitrification and microbial activity were studied with a sequencing batch reactor (SBR)^37^. This study explores multiple mechanisms for the adsorption of Atrazine onto doped and functionalized AHEX graphene quantum dots, including adsorption on surfaces doped with nitrogen, boron, and sulfur atoms through substitutional methods and adsorption involving AHEX deformation by creating vacancies through carbon atom removal. A comprehensive analysis of adsorption energy, charge transfer, and other electronic properties was conducted to understand the impact of decorated graphene on atrazine adsorption.

## Calculation details

Practical algorithms for solving the Kohn–Sham equations have been integrated into increasingly sophisticated codes, significantly broadening the applicability of density functional theory DFT across diverse scientific disciplines such as earth sciences, molecular biology, and materials science. This advancement has extended beyond academia, facilitating innovative research and development in various industrial sectors^38^. The remarkable success of DFT in explaining material behavior has led to the concept of “materials by design,” allowing for the prediction and creation of new materials with specific desired properties that can surpass conventional materials^39^. This study investigates the efficacy of doped graphene quantum dots in adsorbing and removing Atrazine from aqueous solutions using DFT^40^. The calculations were performed in Gaussian 09^41^, which was implemented at the Molecular Modeling and Spectroscopy Laboratory, Centre of Excellence for Advanced Science, National Research Centre. The studied structure was subjected to optimization with the Becke, 3-parameter, Lee–Yang–Parr B3LYP hybrid functional chosen for its proven accuracy in representing the electronic structure of carbon-based nanosystems. The 3-21G atomic basis set was employed to balance computational efficiency and accuracy^42,43^. This study investigates the adsorption mechanisms of Atrazine on various doped graphene variants, including those doped with boron, nitrogen, and sulfur, (AHEX) with vacancies. A comprehensive analysis of the adsorption capacity of these doped graphene structures is conducted. To identify the optimal adsorption sites, the electronic properties of the atoms in both the AHEX variants and the atrazine molecule are evaluated. This evaluation is performed following DFT optimization before conducting the adsorption calculations. Some Important physical parameters were also calculated upon the optimized structure including; binding energy (Eb), total dipole moment, adsorption energy, Charge transfer, HOMO, LUMO, electronic band gap, Molecular electrostatic potential, Infrared spectra, and UV–Visible Absorption.

## Results and discussion

### Stability of optimized structures

**Fig. 1:**
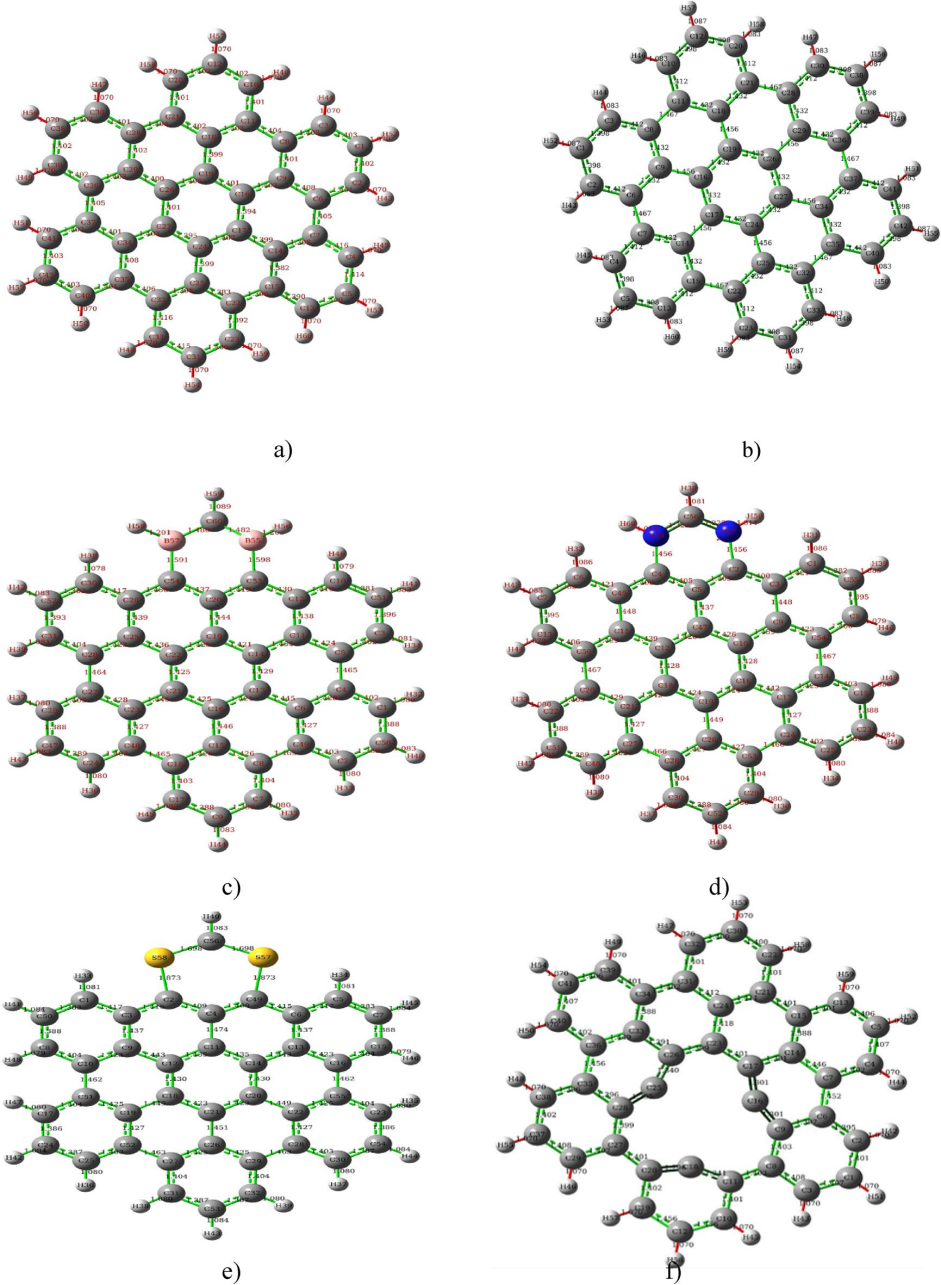
The bond lengths in Graphene quantum dots (AHEX) C–C and C–H bond length (**a**) before and (**b**) after optimization, AHEX boron Doped (**c**) AHEX nitrogen Doped,(**d**) AHEX Sulfur Doped (**e**), and (**f**) AHEX Vacancy.

Gaussian 09 is used to optimize AHEX graphene quantum dots at the B3LYP/3-21G level of computation without symmetry constraints. Figure 1a shows the model molecule used in the study to simulate graphene, which consists of 60 carbon atoms arranged in a hexagonal lattice structure. Subsequently, they investigated different methods of heteroatom doping. Figure 1a, b and Table 2 demonstrate that the carbon–hydrogen bond C–H bond length in AHEX graphene increased slightly after optimization. The bond length changed from 1.07 to 1.086 Å, indicating an overall increase of approximately 1.4%. After optimization, all carbon–carbon bond C–C bonds changed from 1.41 Å in the unoptimized bond to 1.39–1.46 Å in the optimized bond. The bond angle between (C–C–C) is slightly altered in the optimized structure to vary from 118.3 to 121°, with the edge outside having the most open angles.

**Table 2:** Calculated gas phase bond lengths (Å) Range and bond angles (°) of the graphene models.

Structure	d_C–H_ (Å)	d_C–C and C–D_ (Å)	Dopant angles (°) D		
			H–C–D	D–C–D	C–D–C
AHEX (C_42_ H_18_) pristine (D = C)	1.069–1.07	1.3819- 1.416	120.6	118.7	120.16
AHEX (C_42_ H_18_) pristine Opti	1.082–1.086	1.3981–1.466	120	119.9	121
AHEX-B doped (D = B)	1.07- 1.2	1.38–1. 598	121.2–122.4	109.8	121.2
AHEX-N doped (D = N)	1.08	1.31–1.46		129.5	116.1
AHEX-S DOPE D = S)	1.079–1.0842	1.3827–1.8734	115.34	129.3	107
AHEX- acancy	1.07	1.950–1.4561	-		

The C–H, C–C, and C–D bond lengths, and C–C–C, H–C–D, and C–D–C bond angles for pristine unoptimized, optimized, and doped graphene.

Figure 1c–f and Table 2 present the optimized AHEX doped graphene with boron, Nitrogen, and sulfur, focusing on the bond lengths of carbon–carbon (d_C–C_), carbon-hydrogen (d_C–H_), and carbon-dopant bonds (d_D–C_), as well as the bond angles of hydrogen-carbon-dopant, dopant-carbon-dopant, and carbon-dopant-carbon.

Figure 1a indicates that there is no significant change in AHEX graphene structure by optimization in both C–C and C-H bond lengths as well as bond angles. Doped graphene with boron changes the C-B bond length to approximately 1.59 A, which is agreed upon by Cheung^44^. Introducing boron as a dopant into the lattice can induce disorder, particularly when the dopant atoms are positioned adjacently. The bond length in graphene's honeycomb structure is typically shorter than the standard carbon-boron single bond. This structure is attributed to the extensive delocalization of electrons. Moreover, the atomic size of boron is larger than that of carbon.

N-graphene exhibits superior electron transport efficiency compared to pristine graphene^45,46^. In nitrogen-doped graphene, the bond lengths of carbon to nitrogen (C=N) ranged from 1.31 to 1.36 Å, comparable to or slightly lesser than those observed in pristine graphene. This similarity in bond lengths can be attributed to ation of a more stable double bond configuration upon nitrogen doping. Specifically, in the lattice of nitrogen-doped graphene, the incorporation of nitrogen through a pyridinic bonding mechanism, wherein the nitrogen atom is bonded to two carbon atoms located at the lattice edges, showcases an sp^2^ hybridization similar to that of pristine graphene.

Furthermore, in this doped structure, the angle formed by the carbon–doped nitrogen–carbon (C–D–C) sequence is maintained within a range of approximately 116 degrees, preserving the angular geometry characteristic of the hexagonal lattice of pristine graphene^8^ as shown in Fig. 1 d and Table 2. Graphene doped with sulfur exhibits longer bond lengths, typically around 1.87 Å, than pristine graphene. This elongation can be attributed to several factors. When sulfur bonds with carbon in graphene, it forms sp^3^ hybrid orbitals, unlike the sp^2^ hybridization found in pristine graphene. Table 2 compares the characteristics of sp^3^ and sp^2^ hybridization, showing that sp^3^ hybridization results in a more diluted s character (25% s and 75% p) than sp^2^ hybridization. This leads to a tetrahedral geometry with bond angles of approximately 109.5° for sp^3^-hybridized atoms. Additionally, sulfur has a larger atomic radius than carbon, which naturally leads to longer bond lengths when it replaces carbon atoms in the graphene lattice.

The sp^3^ hybridization causes the electron density to spread more, resulting in longer and potentially weaker bonds. These longer bond lengths have significant implications for graphene's properties. The elongated bonds are generally more reactive, which can be benefit for various applications. The change in bond length and hybridization can alter the electronic structure of graphene, potentially opening up new possibilities for tailoring its properties. Furthermore, incorporating sulfur and the resulting longer bonds can introduce local distortions in the graphene lattice, which may affect its mechanical properties.

This bond elongation phenomenon is not unique to sulfur doping. Other dopants with larger atomic radii than carbon also tend to produce longer C–D (carbon–dopant) bonds, with nitrogen being a notable exception due to its similar size to carbon. Understanding and controlling these bond length changes through selective doping offers a promising avenue for modifying graphene's properties for future applications in catalysis, sensing, and electronic devices^47^.

### Binding energy

The computation of the binding energy (E_b_) of doped graphene involves subtracting the ground state energy of the compound system from the total of the ground state energies of the doped atom, carbon, and hydrogen.

$$E_{b} = \left( {\left( {E_{G - D} complex} \right) - \left( {N_{C} E_{C} + N_{D } E_{D} + N_{H} E_{H} } \right)} \right)/N$$

where N_c, D, H,_: number of carbon, dopant, and hydrogen atoms and E_c, D, H_: ground state energy of carbon, dopant, and hydrogen atom, and N is the total atoms per molecule.

**Fig. 2:**
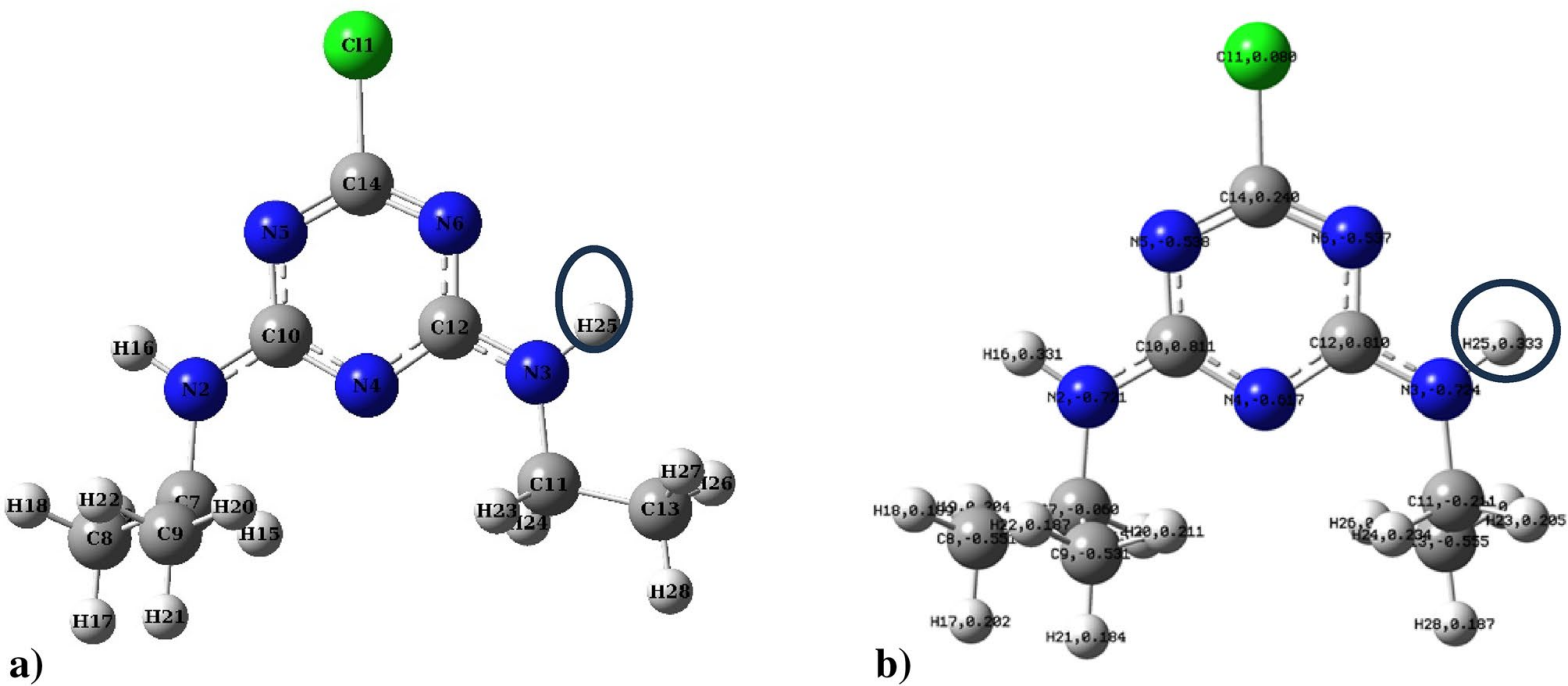
(**a**) Atrazine optimized structure and (**b**) Mulliken charge distribution of atrazine atoms.

The study of binding energies (E_b_) provides crucial insights into the stability of various doped graphene structures. The investigation examined the binding energies of molecules to graphene doped with boron, nitrogen, and sulfur, obtaining values of − 6.92, − 7.19, and − 6.73 eV, respectively. A more negative binding energy indicates stronger binding affinity and better stability of the molecule-graphene system. Nitrogen-doped graphene exhibited the most negative binding energy (− 7.19 eV), suggesting the formation of the most stable complex with the adsorbed molecule. This enhanced stability can be attributed to forming a pyridine-like structure when graphene is doped with nitrogen. The research focused on elucidating interaction mechanisms between Atrazine and various hexagonal graphene (AHEX) forms, including boron-, nitrogen-, and sulfur-doped variants and those with vacancies. Mulliken charge distributions of both AHEX variants and the atrazine molecule were analyzed after optimization using Density Functional Theory (DFT) before adsorption calculations to determine optimal adsorption sites. Figure 2 shows the optimized structure and Mulliken charge distribution of the atrazine molecule, where Fig. 2a depicts the optimized geometry and Fig. 2b illustrates the Mulliken charges on each atom. For boron-doped AHEX, interaction analysis revealed that the terminal hydrogen atom (H25) of Atrazine, with a significant positive Mulliken charge of + 0.333, approaches the carbon atom C60 in the AHEX structure. C60, situated between two boron dopants, exhibits a maximal negative Mulliken charge of − 0.728.

**Fig. 3:**
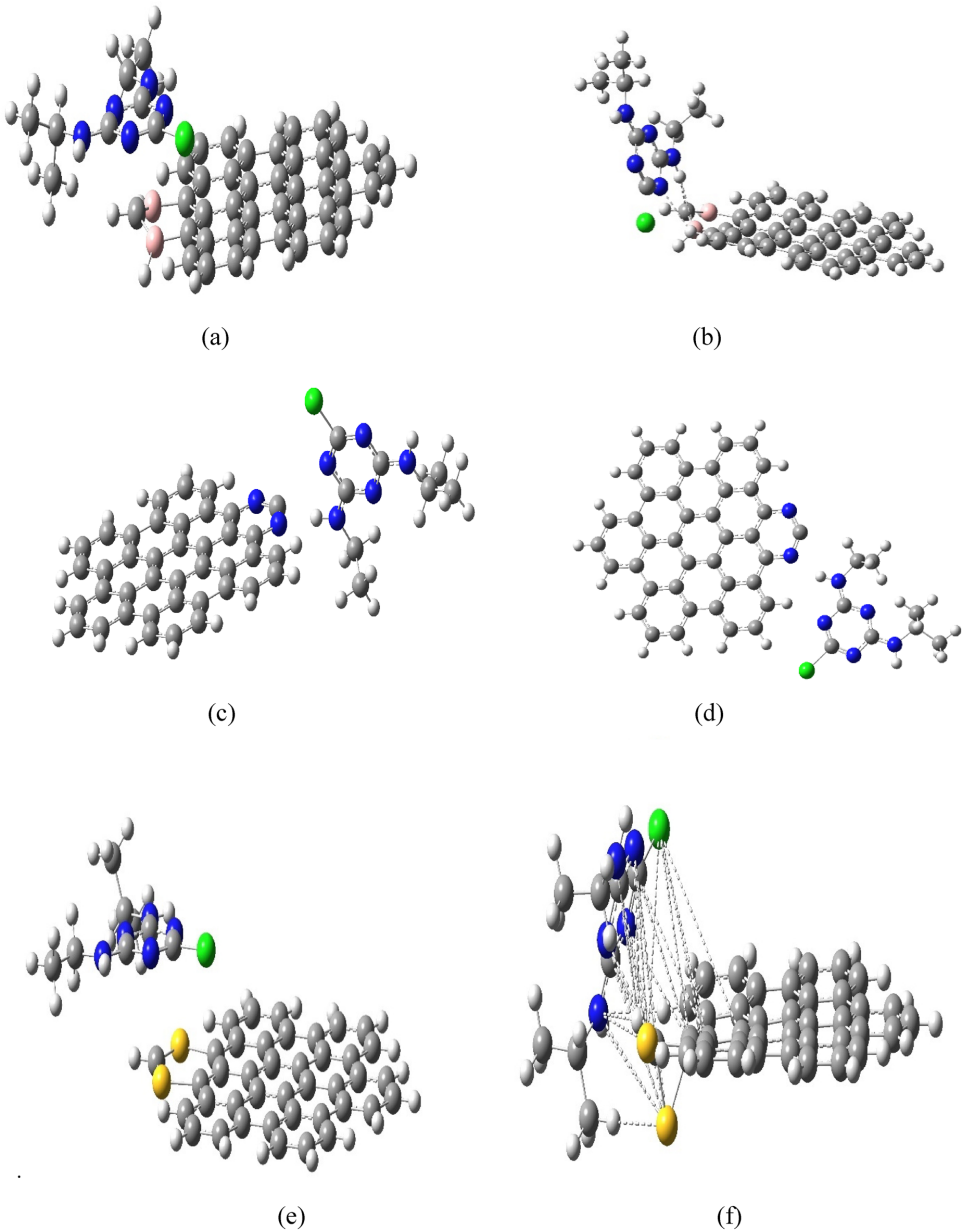
(**a**) Boron-doped AHEX graphene before atrazine adsorption, (**b**) Boron-doped AHEX graphene after atrazine adsorption, (**c**) Nitrogen-doped AHEX graphene before atrazine adsorption, (**d**) Nitrogen-doped AHEX graphene after atrazine adsorption, (**e**) Sulfur-doped AHEX graphene before atrazine adsorption, (**f**) Sulfur-doped AHEX graphene after atrazine adsorption.

Figure 3 presents model molecules of AHEX graphene interacting with Atrazine before and after calculations as part of the research aimed at exploring the interaction mechanisms between Atrazine and different forms of hexagonal graphene (AHEX), including boron-, nitrogen-, and sulfur-doped variants. This proposed configuration facilitates adsorption through an edge-oriented approach at a distance of 3.7 Å from the AHEX surface, as in Fig. 3a. Figure 3b illustrates the adsorption mechanism of Atrazine on boron-doped AHEX graphene, where the edge of the atrazine molecule approaches the AHEX surface. Introducing boron atoms into the hexagonal carbon lattice of graphene significantly alters its electronic structure and chemical properties, leading to enhanced interactions with Atrazine. For nitrogen-doped AHEX, the adsorption mechanism involves the edge of the atrazine molecule approaching the AHEX surface, as in Fig. 3c. The interaction targets nitrogen-doped atoms with the highest negative Mulliken charge of − 0.631. The positively charged hydrogen atom H25, with a charge of + 0.333, is expected to interact with the negatively charged nitrogen atoms of the triazine ring at a distance of 1.92 Å. Figure 3d illustrates the nitrogen-doped AHEX graphene after atrazine adsorption, elucidating the distinctive adsorption mechanism that involves the edge of the atrazine molecule approaching the AHEX surface. Incorporating nitrogen atoms into the graphene lattice significantly modifies its electronic structure and surface chemistry, enhancing interactions with atrazine molecules. This phenomenon can be attributed to the localized positive charges and increased electron density around the nitrogen sites, which create favorable adsorption sites for atrazine molecules on the nitrogen-doped AHEX graphene surface. Figure 3e explores Sulfur-doped AHEX, where the interaction focuses on sulfur atoms within the AHEX framework, which display the highest positive Mulliken value of + 0.958. The adsorption process involves S-triazine nitrogen atoms (N5 and N6) of Atrazine, bearing the most negative Mulliken values of − 0.538 and  − 0.537, respectively. This charge distribution drives the interaction between sulfur-doped AHEX and the Atrazine molecule. Figure 3f illustrates the sulfur-doped AHEX graphene after atrazine adsorption, providing valuable insights into the unique interaction mechanism between Atrazine and the sulfur-doped graphene surface. Incorporating sulfur atoms into the AHEX framework significantly alters graphene's electronic structure and surface properties, enhancing adsorption capabilities toward atrazine molecules.

These elements collectively strengthen the scientific validity of the study, providing a solid foundation for the conclusions drawn about adsorption mechanisms in doped graphene systems.

### Chemical reactivity and adsorption process

#### Total dipole moment and adsorption energy

The reactivity of AHEX is affected by doping through the alteration of the electronic density of states and symmetry. Reactive sites are often associated with regions of high electron density or the presence of localized states. Thus, doped graphene exhibits enhanced chemical reactivity compared to pristine graphene. The TDM of AHEX has a zero value of 0.0 Debye due to the symmetry of the structure. Boron-doped AHEX, where Boron is an atom with fewer valence electrons than carbon, induces p-type doping by introducing holes. This structure disrupts electronic symmetry, creating localized states near the Fermi level and altering electronic state distribution. A notable total dipole moment (TDM) of 8.22 debye, as shown in Table 3, signifies robust p-type doping and enhances the reactivity via hole presence.

**Table 3:** The electronic properties of compounds, namely dipole moment, energy gap, adsorption energy, and charge transfer.

Compound	Dipole moment (Debye)	Adsorption E (eV)	Band gap (eV)	Q (e)
Atrazine	5.89	–	5.99	–
AHEX C42 H18	0.0	–	3.58	–
AHEX-BORON doped	8.22	–	1.30	0.03
AHEX-BORON-ATZ	8.99	0.52	1.54	–
AHEX-NITROGEN	5.29	–	3.45	–
AHEX-NITROGEN-ATZ	4.13	0.62	3.24	–0.018
AHEX-SULFUR	4.14	–	0.29	–
AHEX-SULFUR-ATZ	3.67	2.97	2.90	0.032

On the other hand, Nitrogen, with one more valence electron than carbon, can create n-type doping (introducing electrons); nitrogen changes the local symmetry and introduces states near the Fermi level but on the electron side with TDM 5.29 debye. Sulfur atom size causes significant lattice strain and local potential differences when it substitutes a carbon atom in graphene. The altered TDM (4.14) Debye indicates a moderate impact on the electronic structure compared with nitrogen or boron doping, but it could still modify the reactivity and other properties. Creating a vacancy in graphene somewhat changes the electronic character, but not nearly as much as adding new elements does. There is a modification in reactivity, but it's much less dramatic than doping 0.1 debye.

The adsorption characteristics of a system comprising an adsorbent and an adsorbate are substantially influenced by factors such as the specific surface plane, the nature of the atomic termination at the surface, and the molecule's orientation upon adsorption. The adsorption energy, which represents the energy released when two materials bind together during the adsorption process, is a measure of the interaction strength where an atom, ion, or molecule—the adsorbate—becomes attached to the solid surface of the adsorbent.

The adsorption energy calculation is as follows:

$${E}_{adsorbtion}=(\left({E}_{G-D}+{E}_{Atz}\right)-({E}_{complex}))$$

where ${E}_{G-D}$: is the total energy of the Doped AHEX; ${E}_{Atz}$: is the total energy of optimized Atrazine; ${E}_{complex}$: is the total energy of the complex after adsorption.

Table 3 and Fig. 3a–f show the predicted positive adsorption energy (E_a_). The calculated values validate the robust stability of the adsorption process of Atrazine on doped graphene by boron, nitrogen, and sulfur. Values of adsorption energy show 0.52, 0.62, and 2.97 eV, respectively, but unsatisfied adsorption of Atrazine in vacancy.

#### Charge transfer

The variations in charge density have been determined by comparing the electron density of Atrazine when it is part of the complex post-adsorption to its electron density before adsorption. The charge present on Atrazin following adsorption has been computed using the formula $Q={Q}_{total}-{Q}_{n}$, where Q _total_ represents the charge of Atrazin within the complex after adsorption, and Q_in_ denotes the charge of Atrazin before the adsorption.

Investigating charge density differences provides crucial insights into the electron transfer dynamics between Atrazine and doped graphene substrates. Calculations reveal electron density variations upon adsorption, indicating specific interaction mechanisms between Atrazine and the boron, nitrogen, and sulfur-doped AHEX graphene. Positive charge changes on Atrazine (+ 0.0009 and + 0.032, respectively) demonstrate a net electron migration from the adsorbate to the substrate for boron- and sulfur-doped graphene. This electron transfer underscores the nature of the interaction, with the doped graphene acting as an electron acceptor. The process is accompanied by geometric adjustments in the atrazine molecule, particularly involving the nitrogen atom (N6) in the s-triazine ring and the hydrogen atom (H19), as evidenced in Fig. 3a–f. The observed decrease in negative charge distribution around N6 and the corresponding increase in positive charge on H25 in both doped graphene complexes suggest the presence of hydrogen bonding and π–π interactions. These interactions play a crucial role in stabilizing the adsorption complex. The high electron density at the N6 position in Atrazine's s-triazine ring, characterized by a Mulliken charge of − 0.722, predisposes this nitrogen atom to donate electrons to the sulfur-doped graphene, which exhibits a complementary positive Mulliken charge of + 0.722. In the case of sulfur-doped graphene, the charge complementarity between the electron-rich nitrogen of Atrazine and the electron-deficient sulfur-doped sites facilitates charge transfer.

Additionally, the chlorine atom in Atrazine likely participates in physical bonding interactions, possibly of a Van der Waals nature, with the partially negatively charged carbon atoms adjacent to the sulfur dopant. These multiple interaction points contribute to the overall stability of the adsorption complex. Interestingly, the AHEX-nitrogen-doped scenario presents a reversal in the direction of electron transfer. Here, electrons move from the AHEX-nitrogen-doped graphene to Atrazine, as indicated by a charge change value of − 0.018. This reversal is facilitated by geometric adjustments in Atrazine that align the highly positively charged H25 atom with the negatively charged nitrogen dopant (Mulliken charge − 0.63) in the graphene substrate. This alignment suggests an electron-donating interaction from the nitrogen-doped graphene to the electron-deficient regions of Atrazine, enhancing the adsorption process through complementary charge interactions.

These findings highlight the complex nature of electron transfer in doped graphene-adsorbate systems and demonstrate how different dopants can fundamentally alter the direction and magnitude of charge transfer. Understanding these mechanisms is crucial for optimizing graphene-based materials for specific environmental remediation and sensing technologies applications.

#### HOMO, LUMO, and electronic band gap

The reactivity of molecules in chemical processes is significantly influenced by the characteristics of the highest occupied molecular orbital (HOMO) and the Lowest Unoccupied Molecular Orbital (LUMO). The HOMO represents the electron donor capability of a molecule, where a higher HOMO energy level indicates a greater propensity for electron donation, thus enhancing susceptibility to electrophilic attack. Conversely, the LUMO signifies the electron acceptor potential, with a lower LUMO energy level suggesting an increased tendency to accept electrons, making the molecule more prone to nucleophilic attack. The energy gap between a molecule's HOMO and another's LUMO is a critical determinant of reaction likelihood, with a smaller gap facilitating electron transfer and, thus, reaction occurrence. In the pristine AHEX framework, the observed band gap stands at 3.58 eV. Following the introduction of dopants such as boron, nitrogen, and sulfur, notable decreases in band gap values are recorded to 1.3, 3.45, and 0.29 eV, respectively, as outlined in Table 3. This diminution is linked to forming of new electronic states resulting from doping activities.

**Fig. 4:**
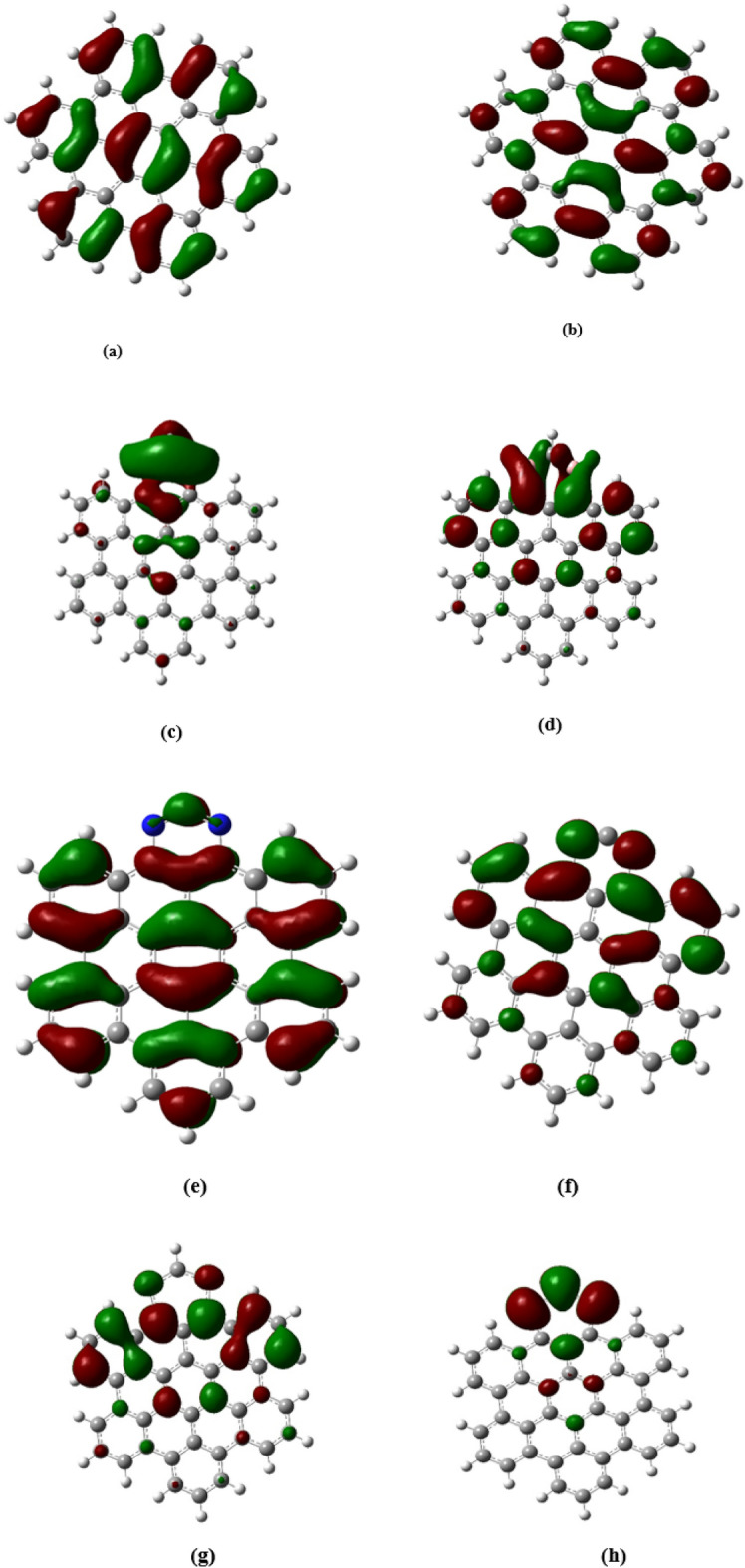
(**a**) HOMO and (**b**) LUMO orbitals of AHEX pristine graphene, (**c**) HOMO and (**d**) LUMO orbitals of AHEX boron-doped graphene, (**e**) HOMO and (**f**) LUMO orbitals of AHEX nitrogen-doped graphene, (**g**) HOMO and (**h**) LUMO orbitals of AHEX sulfur-doped graphene.

Furthermore, an inverse correlation exists between the band gap size and the material's reactivity, indicating that the observed decrease in band gaps due to doping implies an increase in reactivity. The next band gap increases after adsorption of atrazine and Post-atrazine adsorption. The band gaps increased to 1.54 and 2.9 eV for boron and sulfur-doped AHEX, respectively. This result suggests that a larger band gap contributes to the stability of the adsorption complexes, and this results in strong interaction between AHEX-doped graphene and Atrazine due to the passivation of the interactive electrons from atrazine atom, especially in the case of sulfur doping, which has exceptional reactivity when doped with AHEX. Furthermore, the adsorption complex formed with Atrazine is highly stable. In pristine AHEX HOMO is spread out across the graphene sheet, primarily localized around the π-bonds between carbon atoms. This proposal reflects the delocalized nature of the π-electrons in graphene and LUMO uniform potential for accepting electrons, as shown in Fig. 4a, b. HOMO in AHEX- boron doped electron density is shown mostly around the carbon atoms, indicating where electrons are present in the highest occupied molecular orbital. LUMO the electron density clouds are more spread out and cover the boron atoms, as shown in Fig. 4c, d. In HOMO, Areas around the nitrogen atoms exhibit enhanced electron density, illustrating nitrogen’s contribution to the π-system, this is evident from the lobes' size. Additionally, nitrogen introduces localized states.

**Fig. 5:**
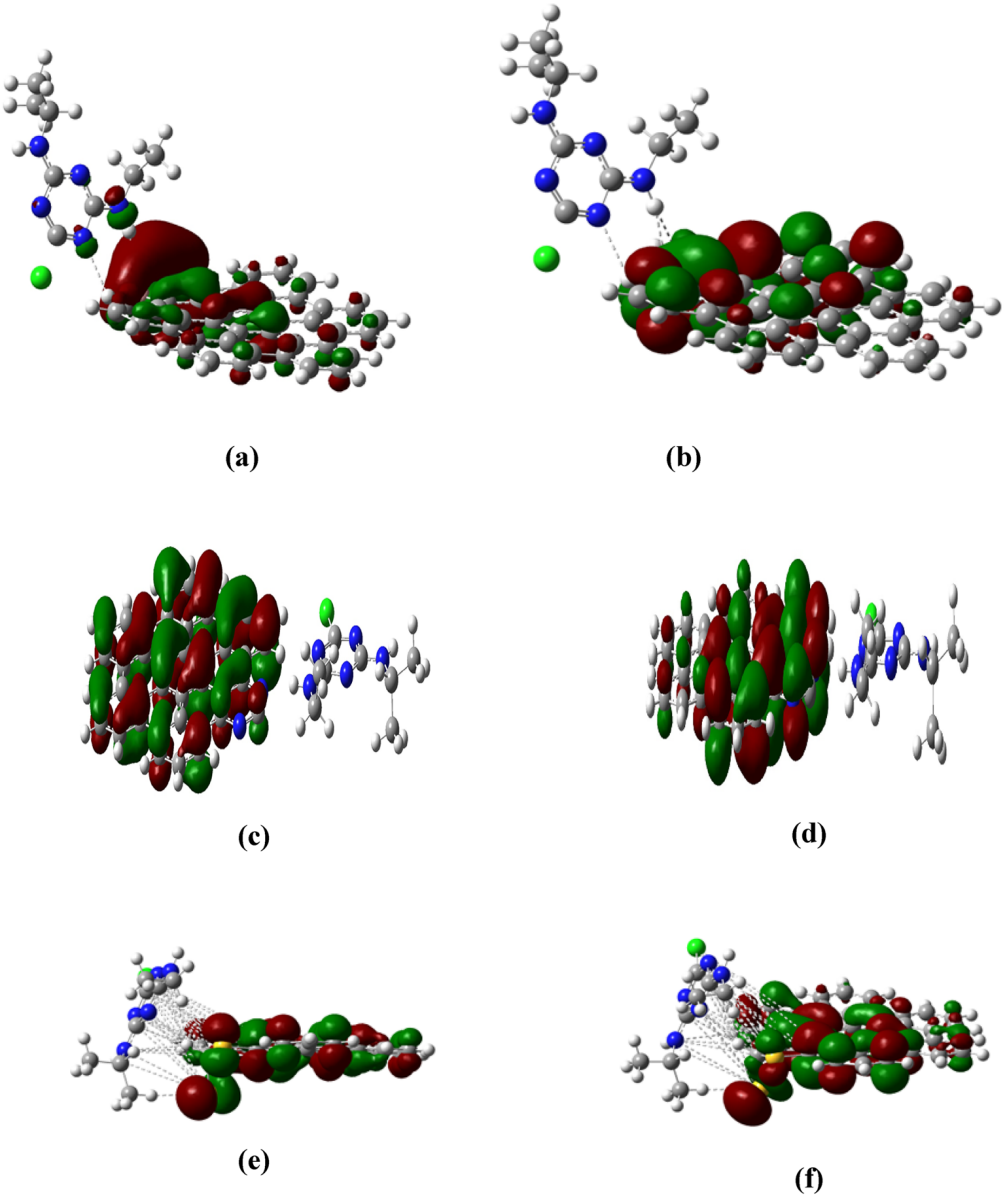
(**a**) HOMO and (**b**) LUMO of AHEX boron-doped graphene after adsorption with Atrazine, (**c**) HOMO and (**d**) LUMO of AHEX nitrogen-doped graphene after adsorption with Atrazine, (**e**) HOMO and (**f**) LUMO of AHEX sulfur-doped graphene after adsorption with Atrazine.

Moreover, a comparison with the electron density distribution in undoped graphene reveals significant changes in the LUMO. Specifically, the LUMO visualization highlights a noticeable shift in electron density toward regions with nitrogen doping, as shown in Fig. 4e, f. The electron density is again localized around the carbon atoms in sulfur-doped AHEX, as shown in Fig. 4g, h, with some density near the sulfur atoms. The electron density for the LUMO is more localized around the sulfur doping sites, which act as electron acceptors. Figure 5a, b illustrates the HOMO and LUMO for the complex formed by Atrazine adsorbed on AHEX-boron doped. The HOMO reveals a prominent red lobe around the boron-doped AHEX, indicating the point of interaction between the atrazine molecule and the graphene surface. This interaction shows electron transfer from Atrazine to graphene, supported by hydrogen bonding with surface atoms and π–π interactions. In Fig. 5c, d Nitrogen displays the HOMO and LUMO configurations for the complex created by Atrazine adsorbed on nitrogen-doped AHEX graphene. The HOMO is predominantly localized across the nitrogen-doped AHEX graphene, indicating a widespread electron distribution.

In contrast, the LUMO is more concentrated around the atrazine adsorption sites. This concentration of the LUMO near the Atrazine proves the transfer of electrons from the graphene to the atrazine molecule. Figure 5e,f illustrates the HOMO and LUMO for the complex formed by Atrazine adsorbed on AHEX-sulfur doped. The HOMO localized around the sulfur atoms in sulfur-doped AHEX. Generally, in complexes, The distribution of the HOMO cubes on the doped AHEX atoms and not on the adsorbed Atrazine implies that Atrazine forms stronger bonds with the AHEX-doped graphene as seen from the HOMO distributions in adsorption complexes Fig. 5a–f.

### Molecular electrostatic potential

The molecular electrostatic potential (ESP) is a theoretical construct describing a molecule's electrostatic effect on a positively charged test particle at various points in space. The ESP helps visualize regions of electron density (negative ESP values, often depicted in shades of red) and electron deficiency (positive ESP values, often depicted in shades of blue) within a molecule.

**Fig. 6:**
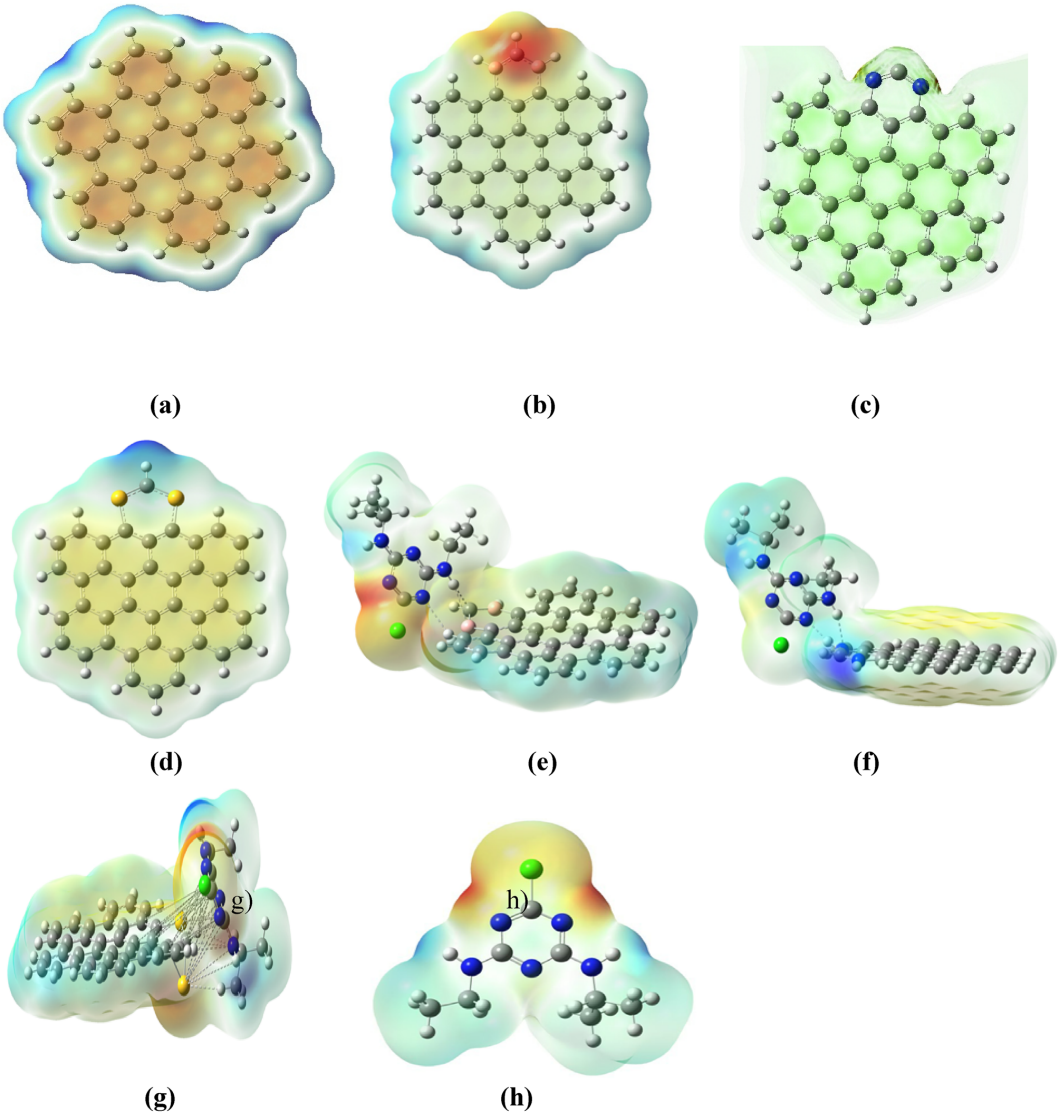
(**a**) ESP of pristine AHEX (**b**) ESP of AHEX-boron doped (**c**) ESP of AHEX doped with Nitrogen (d) ESP of AHEX-sulfur (**e**) ESP of AHEX-boron doped after atrazine adsorption (**f**) ESP of AHEX doped with nitrogen after atrazine adsorption (**g**) ESP of AHEX doped with sulfur after atrazine adsorption (**h**) ESP of Atrazine.

As shown in Fig. 6, the electrostatic potential map of pristine AHEX displays a homogenous pattern, signifying a consistent spread across the observed area. At the same time, in Fig. 6b, AHEX-boron doped doping boron into the AHEX lattice, which carries one fewer valence electron compared to carbon, creates regions of positive potential (blue areas) near the doping sites and red areas around carbon atom between two boron doped atoms in the structure. This proposal suggests that these doping sites could attract nucleophilic species due to a deficit of electron density. Nevertheless, on the other hand, in the case of AHEX, nitrogen-doped nitrogen atoms have one more valence electron than carbon. Nitrogen doping creates areas of negative potential (red patches), which suggests these sites can draw electrophilic species and partially positive charge to atoms adjacent to doped atoms (blue areas), as in Fig. 6c. In AHEX sulfur-doped, the sulfur atoms can contribute lone pairs of electrons (blue areas) and regions of high electron density around carbon. In an ESP map Fig. 6d. After the adsorption process, The Complex of Boron-Doped AHEX with Atrazine, the ESP map undergoes considerable change, revealing novel regions of both positive and negative potential indicating charge transfer between Atrazine and the boron-doped AHEX. Atrazine appears to donate electron density to the boron sites, which is indicated by the shift in the potential around those areas, as shown in Fig. 6e. In the nitrogen-doped AHEX with Atrazine after adsorption, it is clearly shown in the ESP map that the nitrogen-doped AHEX (red) regions as shown in Fig. 6c shifted toward neutral (green to blue color) after the adsorption process as shown in Fig. 6f. In the sulfur-doped AHEX with Atrazine after adsorption, The sulfur atoms in doped AHEX possess vacant electron sites, as shown in Fig. 6f, which convert to reddish, indicating electron transfer from Atrazine to sulfur-doped AHEX.

### Infrared spectra

**Fig. 7:**
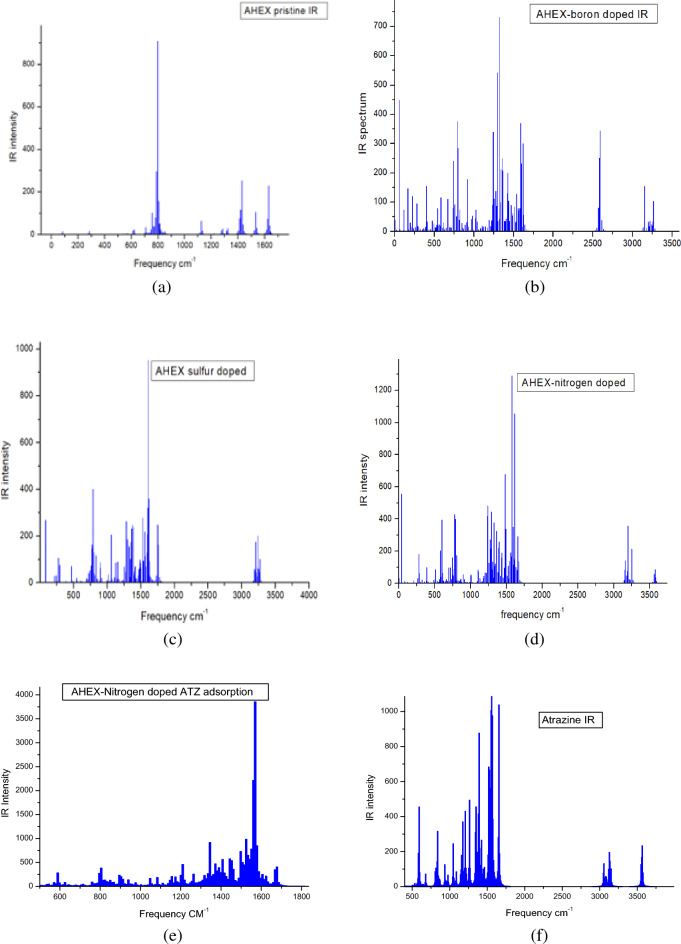
B3LYP/3-21G computed IR spectra of (**a**) AHEX pristine, (**b**) AHEX boron-doped, (**c**) AHEX sulfur-doped, (**d**) AHEX nitrogen-doped, (**e**) AHEX sulfur-doped after adsorption of Atrazine, (**f**) AHEX boron-doped after adsorption of Atrazine, (**g**) AHEX nitrogen-doped after adsorption of Atrazine, and (**h**) atrazine IR spectrum.

Figure 7a, b, c, and d the estimated infrared (IR) spectra using the B3LYP/3-21G method for pristine AHEX and its forms doped with boron, sulfur, and nitrogen are displayed, respectively. These vibrational spectra serve to confirm the stability of previously computed electronic properties, ensuring they are based on optimally structured models. For the boron-doped AHEX, boron–carbon (B–C) bonds are introduced, characterized by their presence in the 1100–1000 cm⁻^1^ range. Meanwhile, the sulfur-doped AHEX reveals a distinct peak at 791.2 cm⁻^1^, indicative of sulfur within a ring structure, a feature absent in the IR spectrum of pristine AHEX. The infrared (IR) spectra of AHEX doped with sulfur, boron, and nitrogen following atrazine adsorption are presented in Fig. 7e, f, and g, respectively. In Fig. 7e, AHEX doped with sulfur shows an increase in peak intensity within the 1000–1400 cm⁻^1^ range, accompanied by slight shifts in peak positions, indicating potential interactions between the adsorbent and atrazine. Figure 7f highlights the IR spectrum of boron-doped AHEX after adsorption, where new peaks emerge in the 1500–2500 cm⁻^1^ range, suggesting the formation of new chemical bonds. Meanwhile, Fig. 7g illustrates the nitrogen-doped AHEX, which exhibits significant changes in both peak pattern and intensity within the 1200–1600 cm⁻^1^ region, further implying strong interactions with atrazine. Additionally, Fig. 7h shows the IR spectrum of pure atrazine, characterized by multiple peaks in the 1000–1500 cm⁻^1^ range, corresponding to C–N stretching vibrations, and peaks above 3000 cm⁻^1^ associated with N–H stretching vibrations. These spectral changes across different dopants underscore the varying degrees of adsorption interactions and chemical modifications induced by atrazine on the AHEX surface.

### UV–visible absorption

Nitrogen edge doping improves the fluorescence properties of graphene quantum dots compared to non-doped structures, causing a blue or red shift in the absorption and emission spectra depending on boundary orbital hybridization. It was also demonstrated that the effect of N-doping is related to the various N-doping patterns and positions, which include pyrazole, pyridazine, graphitic, pyridinic at the center or edge, pyrrolic at the center or edge, and amido at center or edge. Adsorption efficiency is influenced by surface parameters, including porosity, surface area, and functional groups of the adsorbent^48^. Compared to non-doped GQDs, sulfur-doped nanostructures in graphene exhibit a blue shift in the absorption peaks, indicating an increase in S-GQD absorption in the ultraviolet–visible spectrum^49^.

**Fig. 8:**
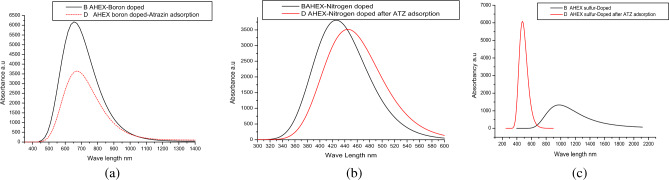
The UV–Vis spectra of (**a**) AHEX-boron doped and AHEX-boron doped/ atrazine complex, (**b**) of AHEX-Nitrogen and AHEX-Nitrogen/atrazine, (**c**) AHEX-Sulfur and AHEX-sulfur/atrazine.

The optical absorption spectra of doped AHEX, before and after adsorption of Atrazine, were analyzed using time-dependent DFT simulations. The analysis considered the first 8 excited states for the AHEX-nitrogen-doped and its complex and three excited states for the optical transitions of boron and sulfur-doped AHEX and their complexes. As shown in Fig. 8a, b, c and Table 4, shows that the prominent absorption peak of AHEX-boron and nitrogen-doped before adsorption at ~ 658.8 nm, 431 nm experience redshift to ~ 676 nm and 444.3 nm) after the adsorption respectively this harmonized with The dominant excitation moved to lower energies following adsorption, in AHEX-boron and nitrogen-doped the energies reduced from 1.88 and 2.87 eV to 1.83 and 2.79 eV respectively. Adsorption of Atrazine on AHEX-boron and nitrogen-doped may increase the concentration of π electrons, resulting in a red-shifted absorption. At the same time, AHEX-boron doped after adsorption of . Atrazine resulted in a notable reduction in the absorbancy of the spectrum, as shown in Fig. 8a.

**Table 4:** Optical parameters for the major and low energy excitations in AHEX-boron, nitrogen, and sulfur-doped before and after adsorption include oscillator strength (f) and wavelength (λ), excitation energy (Ex), excited state (ES), and transition composition (TC).

Structure	f	λ (nm)	Ex (eV)	ES	TC
AHEX-Boron doped	0.0018	2334.71	0.531	S2	H → L 0.70722
	0.0818	658.8	1.8819	S2	H-5 → L − 0.10639
					H-2 → L − 0.19324
					H-1 → L **0.66083(89.9%)**
	0.0708	652.67	1.8997	S2	H-2 → L 0.66887
					H-1 → L 0.19055
AHEX-Boron doped -ATR	0.0024	1575.49	0.787	S2	H → L 0.70072
	0.0741	676.31	1.8333	S2	H-3 → L 0.26138
					H-1 → L **0.64531 (85.9%)**
	0.0161	658.77	1.8821	S2	H-3 → L 0.64698
					H-1 → L − 0.25494
AHEX-Nitrogen-doped (7,8)	0.0641	431	2.8767	S2	H(a)-3 → L + 1 0.11654
					H(a)-1 → L + 1 0.28405
					**H(a) → L 0.64576(42.6%)**
					H(a) → L + 2 − 0.13750
					H(b)-1 → L + 1 0.30369
					H(b) → L 0.59677
	0.031	416.41	2.9775	S2	H(a)-1 → L 0.64531
					H(a) → L + 1 − 0.29591
					H(b)-1 → L 0.64765
					H(b) → L + 1 − 0.26672
AHEX-Nitrogen-doped -ATR (7,8)	0.0145	445.75	2.7815	S2	H(a)-3 → L − 0.19143
					H(a) → L 0.30760
					H(a) → L + 2 0.1186
					H(b)-12 → L + 1 − 0.13326
					H(b)-1 → L + 1 − 0.10394
					H(b)-1 → L + 2 − 0.18475
					H(b) → L − 0.25328
					H(b) → L + 1 0.81972
	0.0627	444.31	2.7905		H(a)-4 → L + 1 0.12178
					H(a)-3 → L 0.10786
					H(a) → L **0.59193(38%)**
					H(a) → L + 2 0.15026
					H(b)-1 → L + 2 − 0.29218
					H(b) → L − 0.49510
					H(b) → L + 1 − 0.44976
AHEX-sulfur-doped	-0.0001	-2745.01	-0.4517 eV	S2	H → L − 0.70704
	0.0308	980.66	1.2643	S2	H-1 → L 0.70378
	0.0022	920.21	1.3473	S2	H-2 → L0.70322
AHEX-sulfur-doped-ATR	0.1095	485.41	2.5542	S2	H-2 → L 0.12165
					**H-1 → L 0.11354(94.2%)**
					H → L 0.67386
	0.0235	460.24 nm	2.6939	S2	H-3 → L 0.25296
					H-2 → L 0.54673
					H-1 → L 0.27384
					H → L − 0.13602
					H → L + 1 0.12012
	0.0244	445.27	2.7845	S3	H-3 → L − 0.13562
					H-2 → L − 0.25639
					H-1 → L 0.27384
					H → L 0.53477
					H → L 0.33756

Significant values are in [bold/italics].

The primary absorption peak of Boron-doped AHEX, initially at approximately 658.8 nm, and the excited state with the greatest oscillator strength (f = 0.0818)is the second excited state (S2). This state features several transitions, the most substantial being from H-1 L, contributing 89.9%. Table 4 shows that the lowest excited state in AHEX boron-doped S2 is primarily dominated by the H L transition, indicating an optical band gap of 0.53 eV oscillator strength (f = 0.0018). This value is less than the electronic gap of 1.3 eV. The smaller optical band gap suggests strong interactions between electrons and holes, forming excitonic states within the electronic energy gap. In nitrogen-doped AHEX, initially at approximately 430 nm, the excited state with the greatest oscillator strength (f = 0.0641)is the second excited state (S2). This state features several transitions, the most substantial being from H(a) L, contributing 42.6%. The state represents the optical gap with a value of 2.87 eV, less than the electronic gap of 3.45 eV depicted in Fig. 8b and Table 4.

In sulfur-doped AHEX, significant changes in the absorption spectra occur following the uptake of Atrazine. A new absorption peak appears around 485 nm, in contrast to the pre-adsorption spectrum at 980.66 nm. This pronounced blue shift in absorbance results from the highly reactive nature of sulfur-doped AHEX, which possesses interactive electrons with a low electronic band gap. It is stated earlier that, due to the greater widths of sulfur atoms compared to carbon atoms, graphene can exhibit considerable chemical activity because electrons can be easily polarized. Adding sulfur to the graphene structure causes the bond polarization to rise^50^. This feature facilitates the interaction between sulfur-doped AHEX and Atrazine, forming strong chemical bonds that passivate the interactive electrons, ultimately creating a new, stable complex with a higher electronic band gap post-adsorption and high absorption energy in UV–VIS spectrum and adsorption energy as shown in Fig. 8c.

### Calculated DFT energy

The computed B3LYP/3-21G energies (E) in Hartree and RMS gradient norms (Hartree/Bohr) for assorted AHEX configurations, doped with boron, nitrogen, and sulfur, as well as following Atrazine adsorption, are shown in Table 5. Significant energy changes based on the doping element and adsorption state are observed in the data. For example, the energy of virgin AHEX is − 1602.6 Hartree, whereas the energy of boron-doped AHEX following atrazin adsorption is significantly lower, at − 2618.0 Hartree. For every configuration, the optimization convergence is indicated by the RMS gradient norms.

**Table 5:** Calculated B3LYP/3-21G E(RB3LYP) energy and RMS gradient norm.

Compound	E (Hartree)	RMS gradient norm (Hartree/Bohr)
AHEX pristine	− 1602.6	0.000048
Atrazin	− 1041.8	0.000004
AHEX-Boron-doped	− 1576.1	0.000004
AHEX-Boron-doped-ATZ adsorption	− 2618.0	0.000005
AHEX-Nitrogen-doped	− 1633.8	0.000012
AHEX-Nitrogen-ATZ adsorption	− 2675.7	0.000002
AHEX-sulfur-doped	− 2317.9	0.000077
AHEX-sulfur-ATZ adsorption	− 3359.8	0.000003

## Conclusion

This study investigates the adsorption mechanisms of Atrazine on various doped graphene variants, including boron, nitrogen, sulfur, and armchair hexagonal graphene (AHEX) with vacancies. Doping AHEX graphene significantly enhances electronic properties, increases the dipole moment, reduces the band energy gap, and improves reactivity. The results indicate strong adsorption capacities of the doped graphene, with adsorption energies of 0.52 eV for boron-doped, 0.62 eV for nitrogen-doped, and 2.97 eV for sulfur-doped AHEX graphene. These high adsorption energies suggest robust π-electron interactions and hydrogen bonding mechanisms. Additionally, the electronic structure analysis reveals that boron and sulfur-doped nanographene exhibit increased band gaps after atrazine adsorption, indicating the formation of strong bonds.

Conversely, nitrogen-doped nanoflakes show a decreased band gap due to additional electron contributions from nitrogen. UV–vis spectroscopy supports these findings, with redshifts to lower energies observed in boron and nitrogen-doped samples and a blue shift in the sulfur-doped sample after adsorption. These findings highlight the potential of doped AHEX graphene nanoflakes as effective adsorbents for Atrazine, offering a promising solution for environmental decontamination. The study underscores the importance of heteroatom doping in enhancing the adsorption properties of graphene, making it a viable material for water purification applications.

## Data Availability

The data will be available upon request; contact Medhat A. Ibrahim medahmed6@yahoo.com.
